# Volumetric measurement of polyacrylamide hydrogel injected for breast augmentation using magnetic resonance imaging

**DOI:** 10.3892/etm.2013.1452

**Published:** 2013-12-17

**Authors:** JIEJIE HU, CHUNJUN LIU, LIN CHEN, WENSHAN XING, JIE LUAN

**Affiliations:** Department of Aesthetic and Plastic Breast Surgery, Plastic Surgery Hospital, Peking Union Medical College, Chinese Academy of Medical Sciences, Beijing 100144, P.R. China

**Keywords:** polyacrylamide hydrogel, volumetric measurement, magnetic resonance imaging

## Abstract

Volumetric measurement of polyacrylamide hydrogel (PAHG) is useful for surgical planning. It is not only a significant factor in the preoperative evaluation of breast augmentation, but may also directly affect the postoperative shape of the breast. The objective of the present study was to evaluate whether magnetic resonance imaging (MRI) is able to provide precise calculations of injected PAHG volumes. MRI scans of ten randomly selected patients were imported to Mimics software. The volumes of PAHG were obtained following the reconstruction of the injected PAHG. In order to assess the precision and observer independency of the technique, the volumes of PAHG were estimated by three plastic surgeons using this method. No significant differences were identified among the PAHG injection volumes calculated by the three observers (P=0.173). The intra-observer correlation coefficient was 0.964, which indicates the precision and feasibility of this method for calculating the volume of PAHG. The use of MRI in combination with Mimics software to calculate PAHG volumes is likely to be of significant clinical benefit in preoperative surgical planning.

## Introduction

Polyacrylamide hydrogel (PAHG) has been widely used for injection augmentation mammaplasty in Russia, China and Iran for more than two decades (1). Although numerous studies have indicated that PAHG injection for soft-tissue augmentation leads to a good result (1,2), a number of other studies have reported high complication rates following the use of PAHG. Reported adverse effects associated with PAHG injection for breast augmentation include indurations, lumps, hematoma, inflammation, infection, persistent mastodynia, poor cosmetic results, glandular atrophy, gel migration, loss of ability to breastfeed and delayed diagnosis of breast cancer (1,3–8).

Since April 2006, when the China State Food and Drug Administration announced that PAHG was prohibited from production and clinical application in plastic surgery, significant social concern was raised concerning the use of PAHG injections as soft tissue fillers. It was estimated that ~200,000 patients have received PAHG breast augmentation in the last decade (1). Between 2005 and 2012, 407 patients came to the Plastic Surgery Hospital (Beijing, China) for PAHG removal due to indurations, lumps, hematoma, inflammation, infection, persistent mastodynia, poor cosmetic results, gel migration or loss of ability to breastfeed. Removal of PAHG often leads to immediate postoperative breast deformity and a significant challenge facing plastic surgeons involves the restoration of a pair of aesthetically pleasing breasts, in terms of fullness and shape. Implant insertion is an effective option for selected patients. However, the majority of patients underwent the PAHG injections in small clinics and often did not receive information concerning the PAHG, including the volume. Therefore, the selection of suitable implants has been difficult.

It is vital to estimate the precise volume and depth of the PAHG injected for breast augmentation. It is not only a significant factor in the preoperative evaluation of breast augmentation, but may also directly affect the postoperative shape of the breast. Therefore, a reliable method for calculating the volume of the injected PAHG is required. Magnetic resonance imaging (MRI) scans are commonly used to analyze the position of the injected PAHG. Therefore, the development of a rapid and precise method of estimating the volume of PAHG on the basis of MRI scans would be of benefit. The purpose of the present study was to define a volume measurement method. In addition, the reliability and precision of the volume measurement method were monitored.

## Materials and methods

Between 2005 and 2012, 407 patients underwent PAHG removal breast surgery in the Department of Aesthetic and Plastic Breast Surgery of the Plastic Surgery Hospital. Each patient underwent breast MRI pre-operatively. Ten patients that had never had breast surgery prior to the study were randomly selected and enrolled in the study. Clinical characteristics of the patients are shown in Table I. Patient age ranged between 25 and 46 years (mean, 34.1 years). The time between the initial PAHG injection to the removal of the PAHG ranged between 2 and 10 years (mean, 6.6 years). Complications included palpable indurations, masses, pain and psychological problems. The study was approved by the institutional review board of Plastic Surgery Hospital of Peking Union Medical College. Prior to breast MRI, informed consents were obtained either from the patients or the patients’ family.

**Figure 1:**
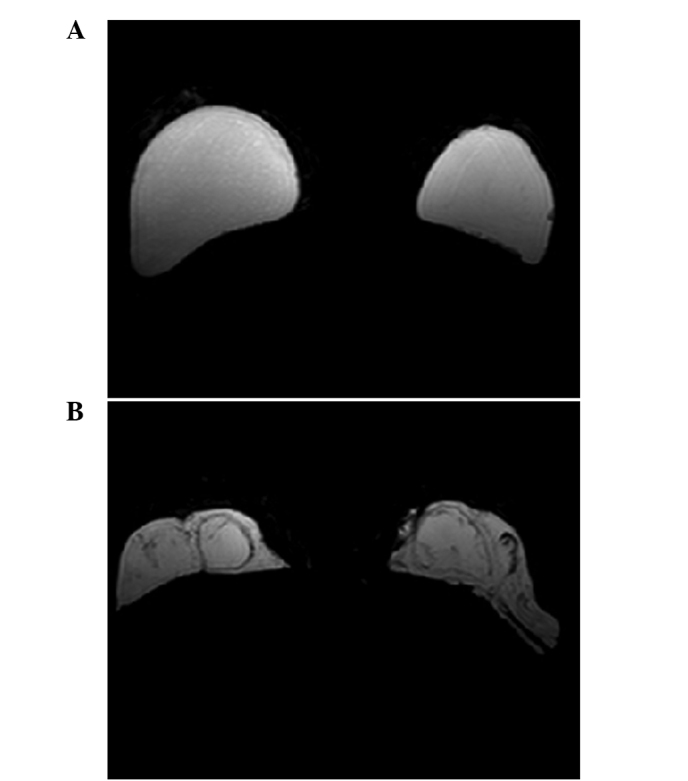
Axial T2-weighted MRI scans with fat depression of PAHG infiltrated in various layers. (A) Patient 8. PAHG was injected into the subglandular space and distributed regularly. (B) Patient 1. PAHG infiltrated into the surrounding tissue and was distributed irregularly. MRI, magnetic resonance imaging; PAHG, polyacrylamide hydrogel.

**Figure 2:**
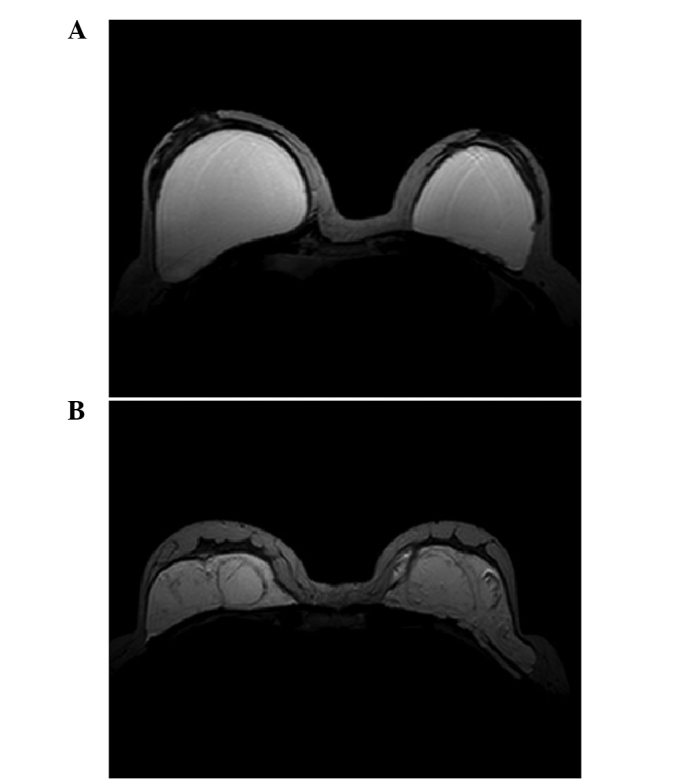
Axial T2-weighted MRI scans without fat depression of PAHG infiltrated in various layers. (A) Patient 8. PAHG was injected into the subglandular space and distributed regularly. (B) Patient 1. PAHG infiltrated into the surrounding tissue and was distributed irregularly. MRI, magnetic resonance imaging; PAHG, polyacrylamide hydrogel.

All patients were scanned prior to the operative procedures by MRI. A 1.5-T MRI scanner (Siemens Magnetom Vision; Siemens AG, Munich, Germany) with dedicated breast coils was used for the imaging. The standard protocol included axial T2-weighted images with and without fat depression and sagittal T1- and T2-weighted images with fat depression. The DICOM images of the MRI scans were imported into Mimics software (Materialise Company, Leuven, Belgium). The axial T2-weighted images with fat suppression (Fig. 1) were used for the reconstruction. On the basis of imaging without fat suppression (Fig. 2), a suitable threshold was selected to label the PAHG. It was necessary to ensure that the colorization threshold was adjusted to label as much of the injected PAHG as possible, while coloring little or no areas around the PAHG. As PAHG has a bright signal, similar to that of water, it may be mistaken for vessels. Based on the T2-weighted imaging without fat suppression, the mislabeled vessels were excluded. Following the imaging, a 3-D reconstruction was performed and the volume of PAHG was calculated using Mimics software.

Three plastic surgeons independently carried out the volumetric measurement. Each patient was measured ten times by three independent plastic surgeons. Calculated PAHG volume data are presented in Table II. The volumes of PAHG injected for breast augmentation were analyzed to calculate the intra- and inter-observer correlation coefficients. Analysis of variance with repeated measurement was performed to analyze inter-observer variance using a global significance level of P<0.05. SPSS version 16.0 for Windows was used for statistical analysis (SPSS, Inc., Chicago IL, USA).

PAHG was injected in different layers and distributed differently in different patients. In patient 8, PAHG was injected into the subglandular space and distributed regularly (Fig. 1A and 2A). In patient 1, PAHG infiltrated into the surrounding tissue and was distributed irregularly (Fig. 1B and 2B).

## Results

Calculated PAHG volume data are presented in Table II. The mean volumes of PAHG injected for breast augmentation ranged between 266.29 and 884.88 ml. No significant differences in the calculated volume of injected PAHG were identified among the three observers (P=0.173). The intra-observer correlation coefficient was 0.964, which indicated high measurement precision and observer independency.

## Discussion

The purpose of the present study was to determine a method for precisely measuring the volumes of injected PAHG. The results demonstrate that the volume of the injected material may be precisely calculated, which is significant for the preoperative evaluation of patients prior to the removal of PAHG. Information concerning the distribution of the injected PAHG and the extent of the tissue infiltration was obtained. It was therefore possible to estimate the difficulty and the risks involved in the removal of the PAHG, as well as ensuring the feasibility of immediate breast augmentation and the postoperative shape of the breast. On the basis of the estimated volume, appropriately sized implants may be selected prior to breast augmentation, following the removal of the PAHG. However, there are specific restrictive indications for immediate breast shape repair. Luo *et al* considered the indications were as follows: Absence of breast neoplasm and infection; removal of >90% of the injected gel; no residual hydrogel in pectoral muscles and/or the subpectoral space; enough healthy mammary tissue and pectoral muscle present to cover the breast prostheses; inframammary folds are intact or are able to be reconstructed simultaneously; and no systemic or psychological problems (9). The present study highlighted additional rules that should be followed. Firstly, the cavity containing the injected PAHG should not be larger than the pocket of the breast implant. Secondly, there should be no residual hydrogel during the closure of the lacunar.

The measurement method may also be used to calculate the volume of the residual hydrogel. The effectiveness of various approaches for the removal of PAHG from the breast may be evaluated on the basis of the estimated volume of residual hydrogel.

There have been few studies analyzing volume measurement of PAHG. However, several studies have discussed the volume estimation of silicone gel-filled breast implants from MRI images (8–12). Previously, Rudolph and Forcier reported a volume measurement method. The authors used MRI plus computer-assisted detection to perform volume calculations (13), which made the calculations more rapid and accurate. However, PAHG breast implants differ from those that are usually confined to capsules. The filler material has been shown to migrate easily, due to muscular activity or the effect of gravity, particularly when the capsules are broken by incorrect massage or incidental force (14). Therefore, it is likely that PAHG implants result in a number of complications, including pain, infection, masses, breast disfigurement and distant migration. The PAHG may also infiltrate into the surrounding tissues and cause degeneration. All traits of PAHG lead to its irregular distribution, which makes the volume measurement more complex. Sun *et al* have reported a method using a 3-D MRI reconstruction technique to determine the volume and distribution of the PAHG (15). However, the study mainly focused on investigating an effective diagnostic method and did not analyze the precision of the measurement method.

In the present study, it was not possible to compare the calculated volumes of injected PAHG with the actual volumes, since information concerning the preoperatively injected PAHG volumes was not available. In addition, it was not possible to compare these volumes with the volume of the removed materials. Firstly, since PAHG is a hydrophilic filler material, some of the material may be easily aspirated following saline dilution. During the surgical procedure, the cavity was repeatedly irrigated with a large quantity of normal saline intraoperatively. Therefore, the amount of the removed PAHG may not be fully consistent with the estimated volume of PAHG. Secondly, the PAHG, as well as the degenerated tissue, were removed intraoperatively leading to the volume of all excised tissue being inconsistent with the estimated volume of PAHG. Thirdly, some of the PAHG may have been injected into another area, including the subcutaneous or intercostal muscles. In consideration of the intraoperative safety and the postoperative shape of the breast, it may not be possible to remove the PAHG completely. This also caused the volume of the materials removed to differ from the calculated volume of PAHG.

In conclusion, MRI imaging offers a precise method for the volumetric measurement of injected PAHG. This is significant for pre- and post-operative evaluation and the selection of implants for immediate reconstruction.

## Figures and Tables

**Table I:** Clinical characteristics of the patients.

Patient	Age, years	Duration of PAHG, years	Complications
1	32	9	Pain
2	45	5	Pain and hardness
3	46	5	Pain and indurations
4	38	10	Psychological problems
5	31	9	Psychological problems
6	29	7	Indurations
7	26	7	Pain, indurations and psychological problems
8	39	8	Indurations
9	30	2	Psychological problems
10	25	4	Masses and psychological problems

PAHG, polyacrylamide hydrogel.

**Table II:** Calculated volumes of PAHG in ml, mean ± SD. Each patient was measured ten times by three independent plastic surgeons.

Parameters	Patient 1	Patient 2	Patient 3	Patient 4	Patient 5	Patient 6	Patient 7	Patient 8	Patient 9	Patient 10
Surgeon 1	417.80±3.80	551.24±3.51	320.57±2.56	265.99±3.15	376.45±3.83	450.07±3.77	499.68±4.23	496.69±3.37	615.84±5.18	881.74±4.52
Surgeon 2	417.65±2.62	552.53±1.41	316.23±1.24	270.15±2.37	374.48±3.67	452.84±5.35	497.97±2.21	495.58±4.20	602.49±3.61	891.81±3.61
Surgeon 3	414.13±3.44	544.64±3.13	319.04±2.87	262.74±2.42	373.95±3.77	452.92±5.20	505.33±2.76	508.23±6.75	603.57±7.74	881.08±4.67
Mean ± SD	416.53±2.08	549.47±4.23	318.61±2.20	266.29±3.71	374.96±1.32	451.94±1.62	500.99±3.85	500.17±7.01	607.30±7.42	884.88±6.01

PAHG, polyacrylamide hydrogel.
